# Supplementary material for: Dynamic arm movements attenuate the perceptual distortion of visual vertical induced during prolonged whole-body tilt
Source: PLoS One. 2021 Apr 30;16(4):e0250851. doi: 10.1371/journal.pone.0250851 (PMC8087117; doi:10.1371/journal.pone.0250851)
Supplement: Attachment — Submitted filename: Reviewer_comment 2 after proofreading.docx [file pone.0250851.s001.docx]

**Reviewer #1**

**Comment 1**

*Restrained subjects were passively tilted to a fixed angle in the coronal plane. The subject’s task was to adjust a display (a line) to align with the “gravitational vertical”. These subjective reports were made before and after arm movements, and after the completion of passive body tilt. In Experiment 2, passive body tilt was ongoing, and subjects were asked to indicate when they felt their body to be aligned with “gravitational vertical”. The results replicated common findings that the subjective vertical can be influenced by passive body tilt. The main finding was that perceived orientation was influenced by arm movements.*

**Responses;**

We appreciate the constructive comments from the reviewers. We have carefully read the comments and corrected our manuscript accordingly.

**Comment 2**
*The authors claim that “no experimental evidence” exists relating active body movement to perception of “gravitational space”. This claim seems odd, given that the authors have cited the work of Bringoux, who studied exactly this topic. In addition, other studies have examined the role of active movement on perception of orientation, in general, and the vertical in particular. Perhaps the closest, with respect to the present study, is the work of Fouque et al. (1999), in which active arm movements were related to whole body tilt in the perception of orientation. In revising, I think the authors should explain how their hypotheses, design, results, and interpretation differ from Fouque et al. In addition, please revise to indicate that the present study provides information only about perception during passive tilt, as contrasted with studies in which body tilt has been actively controlled (e.g., Panic et al., 2015; Riccio et al., 1992). It would be specially helpful, in the Discussion, to consider how future research might help us understand relations between perceived orientation during passive versus active tilt. Achieved orientation can differ from subjective orientation; moreover, outside the laboratory, conscious awareness of orientation is uncommon, whereas (successful) control of orientation is nearly continuous.*

**Responses;**

As pointed out by the reviewer, our description was inappropriate because other papers (Bringoux et al. 2004, 2007; Scotto Di Cesare et al. 2014) have shown the influence of arm movements on spatial judgments even though these results were not positive. Thus, we have removed the description “no experimental evidence” from the manuscript, and accordingly, we have modified the abstract.

Fouque et al. (1999) showed that concurrent arm movements improved accuracy in judgments of the head-referenced eye level, proposing that action promotes spatial judgment. However, this finding cannot tell us whether action also influences the perceptual judgment of gravitational space, not involving body movements. As the CNS would estimate the gravitational vertical by integrating not only sensory inputs but also prior knowledge/experience (Clemens et al. 2011), active body movements may also influence the subsequent perceptual estimates of the gravitational direction via prior knowledge/experience. To assess this hypothesis, we evaluated whether or how active arm movements during prolonged tilt subsequently influenced the perceptual judgments of gravitational direction (SVV or SPV). The results of the during-tilt session showed a significant attenuation of SVV shifts by dynamic arm movements (Fig. 4), at least partially supporting our hypothesis. This result extends Fouque et al.’s finding and suggests that action contributes to perceptual estimates of gravitational space, even without accompanying body movements. We have modified the text (Introduction and Discussion) to emphasize the differences between our study and theirs.

As the reviewer indicates, the body orientation continuously achieved in our daily life would not always be consistent with the conscious and perceived orientation of vertical. Panic et al. (2015) showed that the dissociation of the direction of balance (DOB) from the gravitational vertical influences the achieved body upright during active tilt, but not perceived upright. Unfortunately, since our study used only passive body tilt, it remains unknown whether or how prolonged tilt influences the achieved orientation when actively controlling the body, and whether it is modulated by arm movements. We have described the importance of directly assessing the effects of active body movements on the control of body orientation in future research.

**Changes;**

**Abstract**

Lines 24-28

Concurrent body movements have been shown to enhance the accuracy of spatial judgment, but it remains unclear whether they also contribute to perceptual estimates of gravitational space not involving body movements. To address this, we evaluated the effects of static or dynamic arm movements during prolonged whole-body tilt on the subsequent perceptual estimates of visual or postural vertical.

**Introduction**

Lines 80-94

Previous studies have shown that the body tilt-induced errors in the judgment of the head-referenced eye level considerably decreased when accompanied by arm movements during judgment [32,33]. This finding suggests that active body movements can improve the accuracy of spatial judgments, but it is unknown whether active body movements also influence the perceptual estimates of gravitational space not involving body movements. The CNS considers prior knowledge and experience as well as sensory signals to estimate the gravitational vertical [7-9], allowing us to hypothesize that additional cues generated by body movements may contribute to the subsequent perceptual estimates of the gravitational direction via prior knowledge and/or experience. To test this hypothesis, the present study evaluated whether static or dynamic arm movements during prolonged tilt influenced the perceptual judgments of visual vertical (Experiment 1) or postural vertical (Experiment 2). As mentioned above, the internal estimates of the gravitational direction are distorted during or after prolonged tilt, primarily due to sensory adaptation. We expected that these distorted estimates might be corrected based on additional cues generated by arm movements, resulting in the maintenance of SVV or SPV angles even after prolonged tilt.

**Discussion**

Lines 350-363

Extending previous findings that the accuracy in spatial judgment at the tilted position was considerably improved by accompanying arm movements during judgment [32,33], we hypothesized that active body movements could subsequently influence perceptual estimates of gravitational direction not involving body movements. Based on this hypothesis, we predicted that the perceptual distortion of the gravitational direction induced by prolonged tilt would decrease when active arm movements are performed in the tilt position. In support of our prediction, the results of the during-tilt session showed that the shifts of SVV toward the direction of prolonged tilt (Fig. 3, left panel) were attenuated when the participants performed dynamic arm movements during prolonged tilt (Fig. 4A). Prolonged tilt induces adaptive changes in the vestibular and body somatosensory systems [23,28], leading to a decrease in the sensed angles of the head and/or body relative to gravity [29]. The performance of arm movements against gravity provides supplemental cues such as proprioceptive feedback or efferent copy [30] for estimating head and/or body orientation in space. The CNS would likely recalibrate the internal estimates of the gravitational direction based on these information, resulting in the stable perceptual judgment of visual vertical through prior knowledge/experience.

Lines 399-408

Although the results of the during-tilt session suggest the role of active body movements in the conscious perception of the gravitational direction, it remains unclear whether or how they influence the control of body orientation. Previous studies have shown an inconsistency between perceived and achieved body orientations when actively controlling body orientation [43,44]. This suggests that dynamic body movements may have different effects on the perception of gravitational direction and control of body orientation. On the other hand, some recent studies have demonstrated the contribution of dynamic somatosensory cues to active postural control (Misiaszek et al. 2016, 2017). Future research directly assessing the influence of dynamic arm movements on the achieved body orientation would be helpful for a better understanding of the mechanisms underlying the perception and control of body orientation in space.

**Comment 3**

*It is widely assumed that the body is controlled relative to the direction of gravity, but this view is not universal. In fact, it has come under sustained criticism, mainly because body movement is not constrained directly (or solely) by the gravitational vector but, rather, by the sum of gravitational and inertial forces—the gravitoinertial force vector (e.g.,* *Stoffregen & Riccio, 1988). In the present experiments, the gravitational and gravitorinertial force vectors were the same, and so the results cannot tell us whether participants were responding to one or the other. This limitation of the design should be noted in the Discussion. Similarly, clinical data from stroke patients do not permit the scientist to know which vector is detected.*

**Responses;**

As the reviewer indicates, we used only a static tilt for SVV adjustments, and thus could not dissociate the gravitational and gravitoinertial force (GIF) vectors. Therefore, we cannot conclude whether the perceptual judgments of the gravitational vertical (SVV and SPV) resulted from the responses to either vector. This is a limitation of the present study. As suggested by a number of studies (e.g., Stoffregen & Riccio, 1988), the otolith system responds to the GIF but not solely to the gravitational force; the performance on SVV adjustments would be mainly derived from GIF. On the other hand, a previous study has demonstrated that the estimation of the earth-horizontal direction is influenced differently by whole-body tilt and body centrifugation, even though the GIF vector relative to the head was identical (Carriot et al., 2006). This finding suggests that gravitational force may specifically affect the perception of the gravitational direction. To address this, further studies are needed to dissociate the gravitational and GIF vectors. We have added this description to the text as a limitation of this study. Additionally, we have deleted the description of the relationship between postural control and perception of gravitational vertical in stroke patients from the introduction, since this assumption would not be definitive.

**Changes;**

**Discussion**

Lines 413-421

Second, we used a static whole-tilt for the SVV assessment in which the gravitational and gravitoinertial force (GIF) vectors were the same; therefore, it remains unknown whether participants responded to either force vectors. Since the otolith system responds to both gravitational and inertial forces [28], the visual vertical estimation reflects a response to the GIF. However, a previous study has shown that the estimation of the earth-horizontal direction is differently influenced by whole-body tilt and body centrifugation, even though the GIF vector relative to the head was identical [33]. This implies that the gravitational force may specifically affect the perception of gravitational direction. To address this, further studies are needed to dissociate the gravitational and GIF vectors.

**Comment 4**
*The Introduction should be revised to state explicitly the hypotheses that were tested in the study. What testable predictions did the authors make? Similarly, the Discussion should be revised to re-state the predictions indicating, in each case, whether each prediction was (or was not) confirmed. The pattern of confirmed (vs. not confirmed) predictions should structure data interpretation.*

**Responses;**

We agree with these comments. We had to explicitly state our hypotheses. As we have responded above (please see Comment 2), our hypothesis was that active body movements might also influence the subsequent perceptual estimates of the gravitational direction without body movements. To test this hypothesis, we evaluated whether static or dynamic arm movements during prolonged tilt influenced the perceptual judgments of visual or postural vertical. We predicted that the distorted estimate of the gravitational direction induced by prolonged tilt might be corrected based on additional cues generated by arm movements. Supporting our prediction, in the during-tilt session, the shifts of SVV were significantly attenuated by dynamic arm movements. We have revised the introduction and discussion sections for the readers’ better understanding of the aims and hypotheses of this study and our interpretation of the results.

**Changes;**

**Introduction**

Lines 80-94

Previous studies have shown that the body tilt-induced errors in the judgment of the head-referenced eye level considerably decreased when accompanied by arm movements during judgment [32,33]. This finding suggests that active body movements can improve the accuracy of spatial judgments, but it is unknown whether active body movements also influence the perceptual estimates of gravitational space not involving body movements. The CNS considers prior knowledge and experience as well as sensory signals to estimate the gravitational vertical [7-9], allowing us to hypothesize that additional cues generated by body movements may contribute to the subsequent perceptual estimates of the gravitational direction via prior knowledge and/or experience. To test this hypothesis, the present study evaluated whether static or dynamic arm movements during prolonged tilt influenced the perceptual judgments of visual vertical (Experiment 1) or postural vertical (Experiment 2). As mentioned above, the internal estimates of the gravitational direction are distorted during or after prolonged tilt, primarily due to sensory adaptation. We expected that these distorted estimates might be corrected based on additional cues generated by arm movements, resulting in the maintenance of SVV or SPV angles even after prolonged tilt.

**Discussion**

Lines 350-363

Extending previous findings that the accuracy in spatial judgment at the tilted position was considerably improved by accompanying arm movements during judgment [32,33], we hypothesized that active body movements could subsequently influence perceptual estimates of gravitational direction not involving body movements. Based on this hypothesis, we predicted that the perceptual distortion of the gravitational direction induced by prolonged tilt would decrease when active arm movements are performed in the tilt position. In support of our prediction, the results of the during-tilt session showed that the shifts of SVV toward the direction of prolonged tilt (Fig. 3, left panel) were attenuated when the participants performed dynamic arm movements during prolonged tilt (Fig. 4A). Prolonged tilt induces adaptive changes in the vestibular and body somatosensory systems [23,28], leading to a decrease in the sensed angles of the head and/or body relative to gravity [29]. The performance of arm movements against gravity provides supplemental cues such as proprioceptive feedback or efferent copy [30] for estimating head and/or body orientation in space. The CNS would likely recalibrate the internal estimates of the gravitational direction based on these information, resulting in the stable perceptual judgment of visual vertical through prior knowledge/experience.

**Comment 5**
*Please revise so that the Results of Experiment 1 are presented before the Method of Experiment 2. That is, completely present Experiment 1, and then completely present Experiment 2.*

**Responses;**

According to the reviewer’s suggestion, we have revised the text.

**Reviewer #2**

**Comment 1**

This interesting paper investigates dynamic arm movements as a new variable in the long search for the sensory determinants of the subjective vertical. The 'During Tilt' portion of Experiment 1 first re-demonstrates the known ability of prolonged tilt to bias the subjective visual vertical (SVV) towards the tilt, and then shows (as a new finding) that a series of dynamic arm movements during the tilt reduces this bias. The 'Post Tilt' portion of Experiment 1 shows that this bias reduction does not occur if the SVV is estimated after the participant is moved to a different tilt angle. This may be due to the timing between the prolonged tilt and the SVV estimation (as mentioned by the authors) but could also be due to the new tilt angle 'overwriting' the participant's sense of orientation. Experiment 2 shows that a subjective postural vertical estimation is not affected by the dynamic arm movements in the same manner as the SVV estimation is. The sample sizes are on the small side, as noted by the authors themselves, but the data analysis appears to be well done. I recommend some revisions as follows:

**Responses;**

We are deeply thankful for the reviewer’s helpful comments and advice. We have responded to all the comments and modified the text accordingly.

**Comment 2**

*Lines 46-92*

*The introduction is not as thorough as I would expect in an otherwise well-written paper. Many of the references are grouped together with somewhat superficial descriptions, such as in Lines 49-51. There are very few references from the past 10 years, despite considerable recent research from several labs on the use of dynamic and movement cues on balance. The paper would benefit from a more substantive introduction, which could then be used to deepen the discussion of the results.*

**Responses;**

Several recent studies have shown the involvement of dynamic cues in postural control (e.g., Misiaszerk et al. 2016). However, the purpose of our study was to clarify the mechanism underlying the perception of gravitational direction rather than postural control. As the other reviewer points out (please see #Reviewer 1 Comment 2), the achieved body direction when actively controlling the posture can differ from the perceived orientation. Given this, we have avoided describing the findings of postural control in the Introduction. On the other hand, as the reviewer indicated, our description in the Introduction was too superficial. Therefore, in the new version of the manuscript, we have described the underlying mechanism of the perception of the gravitational direction in more detail based on recent findings, and more explicitly stated our hypothesis in the Introduction. Moreover, we have mentioned the possible effect of arm movements on the control of body orientation based on the findings that dynamic somatosensory cues influence postural control in the Discussion.

**Changes;**

**Introduction**

Lines 46-52

Knowledge of the gravitational direction is fundamental to our action and perception of the earth. The direction of gravity cannot be directly sensed; instead, it is estimated in the brain based on several types of sensory information. Numerous psychophysical studies have demonstrated the involvement of visual [1-3], somatosensory [4-6], and vestibular sensory signals [3,7] in estimates of gravitational direction. Moreover, recent studies using computational modeling have shown that the central nervous system (CNS) weighs and combines these multisensory signals with prior knowledge and experience about the earth-vertical direction in a statistically optimal manner to resolve sensory ambiguity [7-9].

Lines 80-94

Previous studies have shown that the body tilt-induced errors in the judgment of the head-referenced eye level considerably decreased when accompanied by arm movements during judgment [32,33]. This finding suggests that active body movements can improve the accuracy of spatial judgments, but it is unknown whether active body movements also influence the perceptual estimates of gravitational space not involving body movements. The CNS considers prior knowledge and experience as well as sensory signals to estimate the gravitational vertical [7-9], allowing us to hypothesize that additional cues generated by body movements may contribute to the subsequent perceptual estimates of the gravitational direction via prior knowledge and/or experience. To test this hypothesis, the present study evaluated whether static or dynamic arm movements during prolonged tilt influenced the perceptual judgments of visual vertical (Experiment 1) or postural vertical (Experiment 2). As mentioned above, the internal estimates of the gravitational direction are distorted during or after prolonged tilt, primarily due to sensory adaptation. We expected that these distorted estimates might be corrected based on additional cues generated by arm movements, resulting in the maintenance of SVV or SPV angles even after prolonged tilt.

**Discussion**

Lines 399-408

Although the results of the during-tilt session suggest the role of active body movements in the conscious perception of the gravitational direction, it remains unclear whether or how they influence the control of body orientation. Previous studies have shown an inconsistency between perceived and achieved body orientations when actively controlling body orientation [43,44]. This suggests that dynamic body movements may have different effects on the perception of gravitational direction and control of body orientation. On the other hand, some recent studies have demonstrated the contribution of dynamic somatosensory cues to active postural control (Misiaszek et al. 2016, 2017). Future research directly assessing the influence of dynamic arm movements on the achieved body orientation would be helpful for a better understanding of the mechanisms underlying the perception and control of body orientation in space.

**Comment 3**

*Lines 104-106*

*Was there a reason not to secure the participants’ arms by some mechanism that could be loosened/removed at the same time as the display frame was rotated to the left? If the arms were left unsecured then they will necessarily be pulled to the side by gravity when in a static roll, and this would provide an extra, potentially confounding sensory cue. This may have been a reason why the no-movement and static conditions did not significantly differ (Fig 4).*

**Responses;**

Although the participants’ arms should have been restrained, we could not do this methodologically. As indicated by the reviewer, gravity would pull the arms to the side during prolonged tilt, which might provide a cue for the estimation of the gravitational direction. This is another limitation of the present study. We have described this as a limitation in the Discussion section.

**Changes;**

**Discussion**

lines 421-425

Third, the arms were not restrained to the body during prolonged tilt. In such a situation, gravity would have pulled the arms to the side, providing a static cue for the perception of the gravitational direction even when the arm movements were not performed (i.e., No-movement condition). This methodological limitation may be partially responsible for the lack of a significant difference in the SVV angles between the No-movement and Static conditions.

**Comment 3**

*Lines 142-165, Lines 242-253*

*The term ‘trial’ appears to be used for two different types of event: a single instance of the participant setting the white line for SVV (as in line 148), as well as a set of ‘tilt, SVV, task, SVV’ (as in line 153). The explanation of the procedure would be clarified if two different terms were used.*

**Reponses;**

We apologize for the inappropriate writing. To avoid confusion, we have applied the trial “trial” for a single SVV adjustment, and “sequence of experiment trials” for a set of trials in each condition in the new version of the manuscript.

**Changes;**

**Materials and Methods**

Lines 148

Figure 2A shows a sequence of experimental trials during the during-tilt session.

Lines 153-154

The participants performed five trials of the SVV adjustment within 40 seconds.

Lines 156-160

After the display portion was returned to the initial position (i.e., in front of the participant’s face), the shutter opened and the participants were asked to perform the SVV adjustments for five trials again. Each participant performed this sequence of experimental trials for each task condition, that is, 30 trials (three task conditions [No-movement, Static, Dynamic tasks] × 2 phases [before, after task] × 5 SVV adjustments) in total.

Lines 167-168

The shutter opened and the participants were asked to repeat the SVV adjustments for 5 trials.

Lines 171-173

Each participant performed this sequence of trials for each task condition in each final tilt position, i.e. 45 trials [3 task conditions (No-movement, Static, Dynamic tasks) × 3 final tilt positions (0°, ±4°) × 5 SVV adjustments] in total.

**Comment 4**

*Line 159*

*What was the rationale for selecting to test at 4 degrees left, 0 degrees, and 4 degrees right? Would a larger tilt be expected to produce a larger effect?*

**Responses;**

We apologize for this inadequate explanation. Our interest in the post-tilt session was to evaluate how arm movements during prolonged tilt attenuated the after-effect of prolonged tilt on SVV angles near upright. Based on a previous finding showing that approximately 4°is the threshold for the detection of body tilt relative to gravity (Bringoux et al. 2002, *Neuropsychologia*), we assumed that participants could recognize a body tilt at 4°, and used these angles. We have added the reason for the application of such small tilt angles to the text.

The results of the post-tilt session showed that the effect of dynamic arm movements on the SVV shifts tended to be larger (i.e., SVV shifts were more strongly attenuated) at a position closer to the initial tilt position (Fig. 5), probably due to the duration between the SVV and action tasks (as described in line 380-388). Given this, we expect to observe a greater effect of dynamic arm movements at a position (e.g., LSD 8°) closer to the prolonged tilt position.

**Changes;**

**Materials and Methods**

Lines 145-146

These angles were determined based on the fact that 4° is the threshold for the detection of body tilt in the roll plane [35].

**Comment 5**

*Lines 160-162*

*It was not clear to me why the 'Post Tilt' experimental procedure ends with moving the participant to 16 degrees right and then to the start position. These tilts happen after the SVV estimations are made, so are they necessary? If they are necessary for the 'Post Tilt' procedure, why are they not done at the end of the 'During Tilt' procedure?*

**Responses;**

In the post-tilt session, we set three final tilt positions. If the participants were tilted back to the upright position directly from each position after the SVV task, the tilt motion would provide a cue about the final tilt position, which could affect the performance in subsequent trials. To prevent this as much as possible, the participants were returned to the upright position via the RSD 16° position. In the during-tilt session, only one body tilt angle was used; therefore, we did not specifically consider this feedback. If multiple tilt directions and angles were used in the during-tilt session, we inserted a tilt position before returning to upright, as in the post-tilt session. To convey this point more clearly, we have corrected the description of the reason for using RSD 16°in the text.

**Changes;**

**Materials and Methods**

Lines 168-170

After completing the task, the body was returned to the upright position via the RSD 16° position to avoid providing feedback about the final tilt position that could influence the subsequent performance on the SVV adjustment.

**Comment 6**

*Lines 378-382*

*The lack of effect of dynamic arm movements on SVV estimation in the 4 degree Right position for the ‘Post Tilt’ procedure is explained as a result of a small sample size. This may be true, but it may also represent an effect of always using a prolonged left tilt at the start of the experiment. What would happen if the initial tilt was to the right instead?*

**Responses;**

The results in the post-tilt session show a lack of significant effect of dynamic arm movements, not for only RSD 4°, but also for LSD 4° and 0° (please see Fig. 5). However, the effect of arm movements tended to be smaller for the RSD 4° position than for the other positions (although not statistically significant). As indicated by the reviewer, the tilt direction used for prolonged tilt would have contributed to this. The involvement of the additional information generated by arm movements in the internal estimates of the gravitational direction presumably decays with time (as described in lines 380-388). Because the time duration between the SVV and action tasks was longer in RSD 4° than in LSD 4°, the effect of dynamic arm movements may have been less observed in RSD 4° position. We speculate that if the side of prolonged tilt was right, the effect of dynamic arm movements would be larger in RSD 4° than in LSD 4°. We have not specifically stated this because it is speculative. Instead, we have mentioned that the dependency of the effect of dynamic arm movements on final tilt positions may be due to the duration between the SVV and action tasks. In conclusion, we have stated the necessity of evaluating the effect of dynamic arm movements using different tilt directions and angles in the future.

**Changes;**

**Discussion**

Lines 388-390

In favor of this assumption, the attenuation effect of dynamic arm movements on SVV shifts appears to be greater at positions closer to the initial tilt position, where the interval between the action task and SVV adjustment was shorter.

**Conclusion**

Lines 433-438

To provide a comprehensive understanding of the relationship between action and the perception of the gravitational space, we need to further examine how performance in the estimation of the gravitational direction is influenced by the manipulation of temporal (e.g., arm movement velocity, interval between arm movements, and perceptual task) and spatial properties (e.g., direction and angle of arm movements or body tilt).

**Comment 7**

*Figure 1:*

*This figure appears to be added to show how the display rotates in yaw away from the participant. I found it confusing at first, because I was expecting an image showing the roll rotation used in the experiment. Perhaps the figure could be updated to show both of these features.*

**Responses;**

We apologize for the confusing depictions. We have modified the figure to better depict our experimental setup and added a sentence to the figure caption.

**Changes;**

**Figure caption (Fig.1)**

This figure illustrates a situation in which the participants were tilted leftward. The display portion was rotated in yaw, as denoted by a gray arrow.

**Comment 8**

*Figure 3:*

*Why is the SVV angle for the control in the +4 RSD group so different from the controls in the +4 LSD and 0 degrees groups? I would think that all three groups would have very similar values on the control.*

**Responses;**

As pointed out by the reviewer, SVV angles for the Control condition appear to be larger at RSD 4° than at LSD 4° and 0°. However, since our additional analysis showed no significant difference in SVV angles for the Control condition between these positions, we cannot conclude the larger SVV errors specifically for the RSD 4° position. Although we cannot exactly explain the reason for the tendency (not statistically significant), this may reflect the structural and functional properties of otolith organs given that the CNS would weigh more heavily on otolith signals for the SVV adjustments near upright than somatosensory signals (Clemens et al. 2011). Since this assumption is speculative, we have not specifically described this in the text. Instead, we have added the statistical results showing no significant difference in the angle of SVV for the control condition between the final tilt positions.

**Changes;**

**Discussion**

Lines 232-237

For the post-tilt session, the median SVV angles (1st, 3rd quartiles) in the Control and No-movement conditions were -1.0° (-1.5, 0.7) and -2.7° (-6.0, -0.4) for the LSD 4° position, -1.5° (-2.5, 0.5), and -3° (-5.6, -0.9) for the 0° position, and -3.5° (-6.5, -1.5) and -4.0° (-8.6, -1.8) for the 4° RSD position. The SVV angles for the Control condition were not significantly different between the final tilt positions (Friedman test; main effect χ2 = 2.07; p = 0.36).

**Comment 9**

*Figure 3:*

*Why is the variability so much larger for the +4 RSD group, compared to the +4 LSD and 0 degrees groups?*

**Responses;**

The inter-individual variability in the SVV angles appeared to be larger for the RSD 4°position than for the other positions. Unfortunately, as with Comment 7, we cannot explain the reason for this based on our data. The characteristics of the otolith function may also contribute to this. We have not specifically stated this in the text because it is too speculative. We need to address this issue in future studies.
